# The insufficiency of CT examination in early detection of central lung squamous cell carcinoma and squamous epithelial precancerous lesions

**DOI:** 10.1186/s12885-024-12052-9

**Published:** 2024-03-05

**Authors:** Jiaming Zhou, Bijun Yu, Peng Guo, Shi Wang

**Affiliations:** Department of Endoscopy, Zhejiang Cancer Hospital, Hangzhou, China

**Keywords:** CT examination, Lung cancer, Bronchoscopy, Central, Squamous cell carcinoma, CT-occult, Squamous epithelial precancerous lesions

## Abstract

**Background:**

CT examination for lung cancer has been carried out for more than 20 years and great achievements have been made in the early detection of lung cancer. However, in the clinical work, a large number of advanced central lung squamous cell carcinoma are still detected through bronchoscopy. Meanwhile, a part of CT-occult central lung squamous cell carcinoma and squamous epithelial precancerous lesions are also accidentally detected through bronchoscopy.

**Methods:**

This study retrospectively collects the medical records of patients in the bronchoscopy room of the Endoscopy Department of Zhejiang Cancer Hospital from January 2014 to December 2018. The inclusion criteria for patients includes: 1.Patient medical records completed, 2.Without history of lung cancer before the diagnosis and first pathological diagnosis of primary lung cancer, 3.Have the lung CT data of the same period, 4.Have the bronchoscopy records and related pathological diagnosis, 5.The patients undergoing radical surgical treatment must have a complete postoperative pathological diagnosis. Finally, a total of 10,851 patients with primary lung cancer are included in the study, including 7175 males and 3676 females, aged 22–98 years. Firstly, 130 patients with CT-occult lesions are extracted and their clinical features are analyzed. Then, 604 cases of single central squamous cell carcinoma and 3569 cases of peripheral adenocarcinoma are extracted and compares in postoperative tumor diameter and lymph node metastasis.

**Results:**

115 cases of CT-occult central lung squamous cell carcinoma and 15 cases of squamous epithelial precancerous lesions are found. In the total lung cancer, the proportion of CT-occult lesions is 130/10,851 (1.20%). Meanwhile, all these patients are middle-aged and elderly men with a history of heavy smoking. There are statistically significant differences in postoperative median tumor diameter (3.65 cm vs.1.70 cm, *P* < 0.0001) and lymph node metastasis rate (50.99% vs.13.06%, *P* < 0.0001) between 604 patients with operable single central lung squamous cell carcinoma and 3569 patients with operable peripheral lung adenocarcinoma. Of the 604 patients with squamous cell carcinoma, 96.52% (583/604) are male with a history of heavy smoking and aged 40–82 years with a median age of 64 years.

**Conclusions:**

This study indicates that the current lung CT examination of lung cancer is indeed insufficiency for the early diagnosis of central squamous cell carcinoma and squamous epithelial precancerous lesions. Further bronchoscopy in middle-aged and elderly men with a history of heavy smoking can make up for the lack of routine lung CT examination.

## Background

Primary lung cancer, with its morbidity and mortality, occupies the first place among malignant tumors worldwide [1]. Relevant study has shown that the 5-year survival rate of stage I and Stage IV lung cancer patients has a huge difference, which is 55.5% and 5.3% respectively [2]. Therefore, the early diagnosis of lung cancer is very important. Currently, Lung CT examination is the main method to detect lung cancer [3]. Since 2005, China has carried out a series of lung cancer screening programs based on lung CT examination, which has made great progress in the early diagnosis of lung cancer [4–5], but the proportion of stage III-IV is still as high as 64.6% in the overall lung cancer [6].

A number of recent international studies have shown that lung CT examination significantly improves the early detection rate of peripheral lung adenocarcinoma [7–10], and more and more nodules with a diameter of less than 5 mm have been found early. However, in the clinical work, a large number of advanced central lung squamous cell carcinoma are still detected through bronchoscopy. Meanwhile, a part of CT-occult central lung squamous cell carcinoma and squamous epithelial precancerous lesions are also accidentally detected through bronchoscopy.

This study reviews the medical records of patients with primary lung cancer who are first diagnosed in the Bronchoscopy room of the Endoscopy Department of Zhejiang Cancer Hospital from January 2014 to December 2018, in order to evaluate whether the current lung CT examination mode is insufficient in the early detection of central lung squamous cell carcinoma and squamous epithelial precancerous lesions and determine which group of people need to undergo further bronchoscopy after lung CT examination.

## Methods

### Study participants

This study retrospectively collects the medical records of patients in the bronchoscopy room of the Endoscopy Department of Zhejiang Cancer Hospital from January 2014 to December 2018. The inclusion criteria for patients includes: 1.Patient medical records completed, 2.Without history of lung cancer before the diagnosis and first pathological diagnosis of primary lung cancer, 3.Have the lung CT data of the same period, 4.Have the bronchoscopy records and related pathological diagnosis, 5.The patients undergoing radical surgical treatment must have a complete postoperative pathological diagnosis. The study is approved by the Ethics Committee of Zhejiang Cancer Hospital. The diagnosis of lung cancer is based on WHO Classification of Lung, Pleural, Thymus and Cardiac Tumors, 4th Edition (2015) [11]. A total of 10,851 patients who meet the study inclusion criteria are included in this study,including 7175 males and 3676 females, aged 22–98 years.

### Study design

This study first describes the general clinical features of CT-occult lesions, and then compares and analyzes the differences in postoperative tumor diameter and lymph node metastasis rate between operable single central squamous cell carcinoma and peripheral adenocarcinoma, in order to demonstrate the insufficiency of the current CT examination for lung cancer in the diagnosis of central squamous cell carcinoma and squamous cell precancerous lesions.

#### Classification of total cases

This study describes the occurrence of different pathological types of lung cancer. Lung cancer can be divided into 13 categories according to the pathological types of the first diagnosis. The double primary lung cancer of different pathological types is a special case, so it is not classified from the first diagnosis tumor, and it is classified into a category. The total of 2809 cases of squamous cell carcinoma are divided into 4 parts including 2227 cases of single central squamous cell carcinoma, 485 cases of peripheral squamous cell carcinoma, 84 cases of double central squamous cell carcinoma and 13 cases of central squamous cell carcinoma with another central squamous precancerous lesion. The number of lung cancers by pathological type from 2014 to 2018 is shown in Table 1.

**Table 1:** Occurrence of various types of lung cancer from 2014 to 2018

Pathological types	Number of different years (n)					
	2014	2015	2016	2017	2018	Total
adenocarcinoma	782	929	1010	1350	1526	5597
Squamous cell carcinoma	615	530	537	567	560	2809
small cell carcinoma	238	273	250	248	219	1228
Cancer/non-small cell carcinoma	210	176	151	166	112	815
Adenosquamous carcinoma	10	16	21	27	18	92
Large cell carcinoma	2	6	8	7	7	30
Large cell neuroendocrine tumors	3	5	10	14	12	44
Mucoepidermoid carcinoma	2	4	0	1	2	9
Lymphoepithelioid carcinoma	0	1	4	1	4	10
Adenocystic carcinoma	2	1	2	2	3	10
Sarcomatoid carcinoma	12	12	15	10	24	73
double primary lung cancer of different pathological types	8	8	14	14	11	55
Single complex cancer	14	9	15	14	27	**79**
Total	1898	1970	2037	2421	2525	10,851

#### CT-occult lesions

A total of 130 CT-occult lesions are found out of 10,851 patients. This study describes the clinical characteristics of these patients. CT-occult central lung squamous cell carcinoma and squamous epithelial precancerous lesions are defined as that cannot be detected by routine lung CT examination.

#### Operable single central lung squamous cell carcinoma and peripheral lung adenocarcinoma

The remaining pathological types of lung cancer are excluded from the total of 10,851 cases, and only the cases of squamous cell carcinoma and adenocarcinoma are counted. These two types of lung cancer are further divided into bronchoscopy confirmed lung cancer group and non-bronchoscopy confirmed lung cancer group. Bronchoscopy confirmed lung cancer is defined as routine bronchoscopy can see the lung cancer lesion, and biopsy and pathological diagnosis can determine the tumor type. It can also be considered as central lung cancer in a broad sense. Non-bronchoscopy confirmed lung cancer is defined as routine bronchoscopy can not see the lung cancer lesion. It can be equated to peripheral lung cancer.

**Fig. 1:**
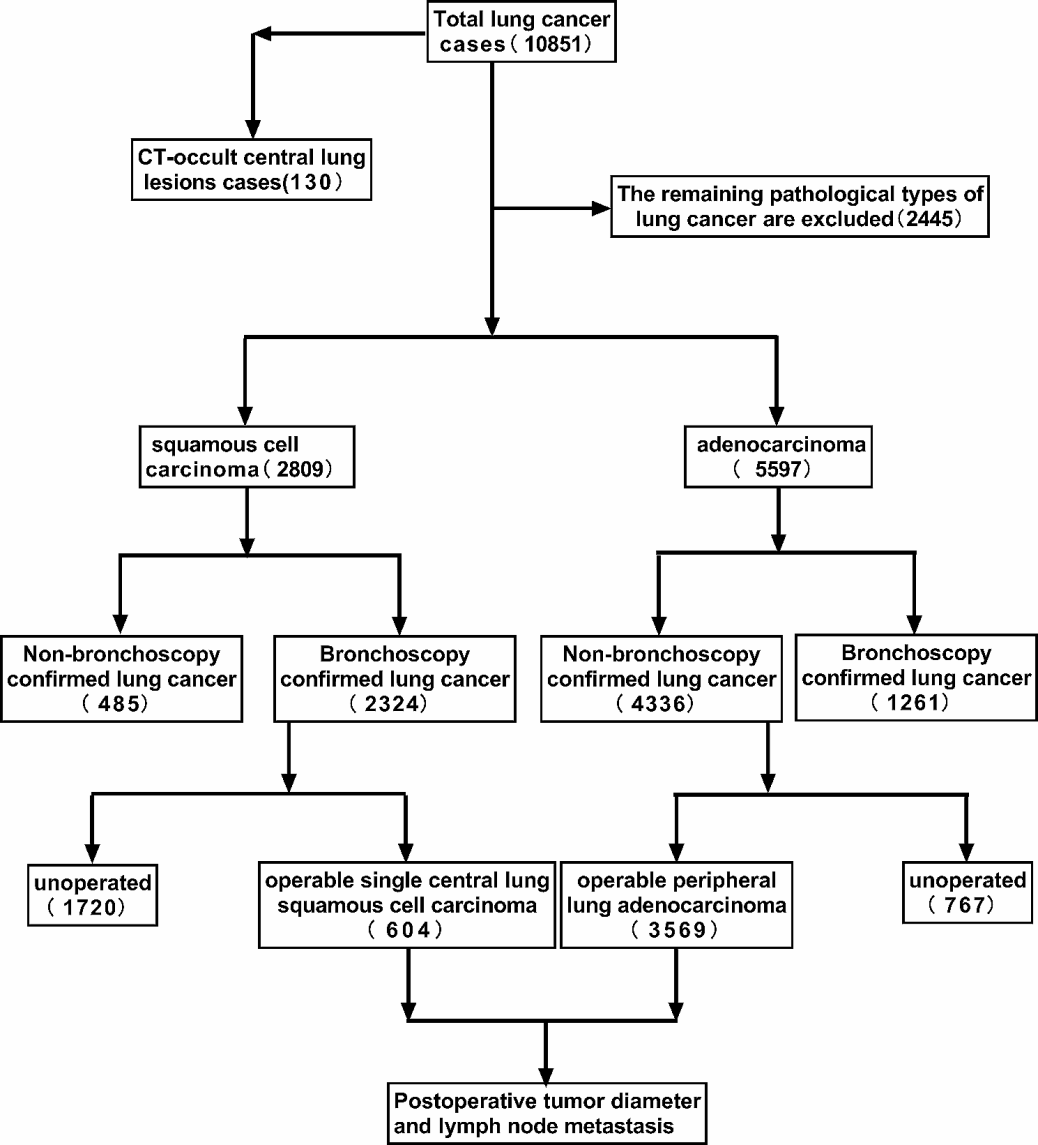
Flowchart of the selection of the patients

Then, from the bronchoscopy confirmed lung cancer group, a total of 604 cases of single central squamous cell carcinoma that could be treated by surgery are screened, while from the non-bronchoscopy confirmed lung cancer group, a total of 3569 cases of peripheral adenocarcinoma that could be treated by surgery are screened. This study will compare the difference in tumor diameter and lymph node metastasis between operable single central lung squamous cell carcinoma and peripheral lung adenocarcinoma.(Fig. 1).

The tumor diameter is closely related to the years of tumor growth. The occurrence of central lung squamous cell carcinoma is a process from dysplasia to carcinoma in situ and then to invasive carcinoma. The size of the tumor diameter can indirectly indicate whether the tumor is diagnosed early. For patients undergoing surgery, such as multiple primary adenocarcinoma lesions during the same operation, only the tumor with the largest diameter is selected for analysis when calculating the diameter of adenocarcinoma.

### Statistical analysis

Statistical software SPSS 22.0 (SPSS Inc. Chicago, IL, USA) is used for data analysis. Qualitative variables are expressed as frequency and proportion and quantitative variables are expressed as the median, maximum and minimum values. The tumor diameter between operable single central lung squamous cell carcinoma and peripheral lung adenocarcinoma is compared by Mann–Whitney U test. The lymph node metastasis between operable single central lung squamous cell carcinoma and peripheral lung adenocarcinoma is compared by X^2^ test. In this study, *P* < 0.05 is considered as statistically significant.

## Results

The 115 cases of CT-occult central lung squamous cell carcinoma and 15 cases of squamous epithelial precancerous lesions are detected. The 15 precancerous lesions include 6 mild squamous epithelial dysplasia, 6 mild to moderate squamous epithelial dysplasia, and 3 moderate to severe squamous epithelial dysplasia. In the total lung cancer, the proportion of CT-occult lesions is 130/10,851(1.20%) (Table 2). In the 55 double primary lung cancer of different pathological types patients, 31 cases of CT-occult central lung squamous cell carcinoma are detected and the first diagnosis of lung tumor in these patients is non-squamous cell carcinoma. The CT-occult central lung adenocarcinoma and small cell carcinoma are not detected. Meanwhile, all these patients are middle-aged and elderly men with a history of heavy smoking.

**Table 2:** CT-occult central lung squamous cell carcinoma and squamous epithelial precancerous lesions

Pathological classification	Total numbers (n)	Number and proportion of CT-occult lesions (n,%)
Double central squamous cell carcinoma	84	84(100%)
double primary lung cancer of different pathological types	55	31 (56.36%)
Central squamous cell carcinoma with another central squamous precancerous lesion	13	13 (100%)
Central large cell neuroendocrine tumors with another central squamous precancerous lesion	1	1(100%)
Central sarcomatoid carcinoma with another central squamous precancerous lesion	1	1(100%)
Total Lung cancer	10,851	130 (1.20%)

Double central squamous cell carcinoma: The first discovered central squamous cell carcinoma accompanied by another primary central early squamous cell carcinoma

Different pathological types of double primary lung cancer: The first discovered lung tumor in these patients is non-squamous cell carcinoma accompanied by another primary central early squamous cell carcinoma

**Fig. 2:**
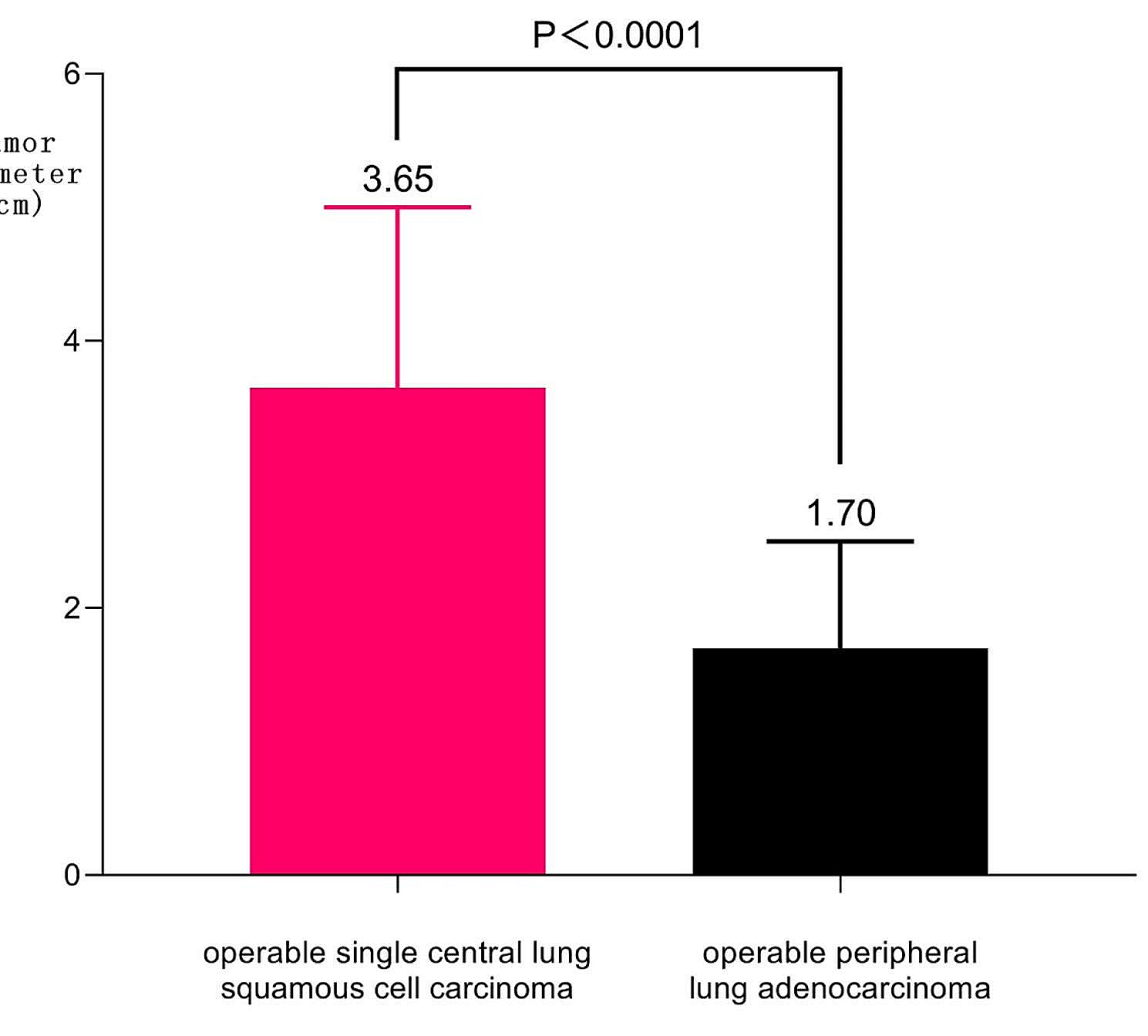
Difference in postoperative tumor diameter between operable single central lung squamous cell carcinoma and peripheral lung adenocarcinoma

The postoperative tumor diameter of operable single central lung squamous cell carcinoma ranges from 0.50 to 14.00 cm, with a median of 3.65 cm. The postoperative tumor diameter of operable peripheral lung adenocarcinoma ranges from 0.40 to 12.00 cm, with a median of 1.70 cm. The postoperative lymph node metastasis rate of operable single central lung squamous cell carcinoma is 50.99% (308/604). The postoperative lymph node metastasis rate of operable peripheral lung adenocarcinoma is 13.06% (466/3569). There are statistically significant differences in postoperative median tumor diameter (*P* < 0.0001) and lymph node metastasis rate (*P* < 0.0001) between 604 patients with operable single central lung squamous cell carcinoma and 3569 patients with operable peripheral lung adenocarcinoma. Of the 604 patients with squamous cell carcinoma, 96.52% (583/604) are male with a history of heavy smoking and aged 40–82 years with a median age of 64 years. (Fig. 2; Table 3)

**Table 3:** Comparison of surgical results of single central lung squamous cell carcinoma and peripheral lung adenocarcinoma

	Single central squamous cell carcinoma	Peripheral adenocarcinoma	*P* Value
	*n* = 604	*n* = 3569	
Number (%)			
Number of postoperative pulmonary lymph node metastases	308/604(50.99%)	466/3569(13.06%)	*P* < 0.0001

The proportion of patients with lymph node metastasis: the number of patients with lymph node metastasis/the number of patients undergoing operations

## Discussion

To our knowledge, this is the first study to evaluate the insufficiency of lung CT alone in the diagnosis of central lung squamous cell carcinoma and squamous epithelial precancerous lesions based on actual data from lung cancer patients. In this study, 130 cases of CT-occult lesions are found, and the data indicate that CT examination is insufficient in early detection of central airway lesions. Meanwhile, the data of this study show that operable peripheral adenocarcinoma is significantly better than single central squamous cell carcinoma in terms of postoperative tumor diameter and lymph node metastasis. These results indicate that compared with peripheral adenocarcinoma, the current lung CT examination has some insufficiency in the early diagnosis of central squamous cell carcinoma.

The increase in the number of lung cancer diagnoses between 2014 and 2018 is due to an increase in the detection of peripheral lung adenocarcinoma in this study. The number of squamous cell carcinoma diagnoses remains relatively stable. These data show that current lung CT examination has a significant advantage in the early diagnosis of peripheral lung adenocarcinoma (Table 1). Relevant international study shows that compared with non-lung CT screening group, adenocarcinoma accounts for the highest proportion of lung cancers detected through screening in the lung CT screening group [12]. Meanwhile, relevant study also shows that the incidence of female lung cancer is increasing year by year [13].

However, central squamous cell carcinoma is closely related to smoking. There are a large number of smokers in China, and central squamous cell carcinoma is still very common. Related study has shown that the common pathological subtypes are adenocarcinoma (74.58%) and squamous cell carcinoma (18.01%) in China. Adenocarcinoma (58.5%) and squamous cell carcinoma (31.6%) are the main pathological types in male patients, while adenocarcinoma (91.6%) and squamous cell carcinoma (3.4%) are the main pathological types in female patients. Adenocarcinoma and squamous cell carcinoma account for 50.6% and 37.7% respectively in smoking patients, the proportion of adenocarcinoma decreases with age, while squamous cell carcinoma and small cell carcinoma increases [14]. Data from another study shows that as one of the most common histologic subtypes of Lung cancer, the overall prevalence of lung squamous cell carcinoma is reported to be approximately 30% [15]. The overall international statistics show that in 2020, there were an estimated 2,206,771 new cases of lung cancer, with 1,435,943 in males and 770,828 in females worldwide. In males, 560,108 (39%) of all lung cancer cases were adenocarcinoma, 351,807 (25%) were squamous cell carcinoma. Most squamous cell carcinoma cases were found in the USA, China, and the UK [16]. Therefore, early diagnosis of squamous cell carcinoma is still of great significance in China.

This study results indicate that compared with peripheral adenocarcinoma, the current lung CT examination has some insufficiency in the early diagnosis of central squamous cell carcinoma(Fig. 2; Table 3). Meanwhile, only 604 of central lung squamous cell carcinoma can be treated with surgery, and even if surgery is performed, patients will lose at least 20% of lung function, which directly affects the quality of life of patients. Related research also shows that lung squamous cell carcinoma accounts for about 20-30% of non-small cell lung cancer. When diagnosed, patients are often older, stage is late, complications are more frequent, and tumors are mostly located in the center, which is difficult to treat. The median survival of patients is about 30% shorter than that of patients with other non-small cell lung subtypes [17]. Related research also shows that although lung squamous cell carcinoma accounts for only 15% of the lung cancer cases, more than 315,000 new cases are diagnosed each year.Squamous cell carcinoma has a poor prognosis with a five-year survival rate of less than 20% for patients with inoperable squamous cell carcinoma and limited treatment options [18]. Only by realizing early diagnosis of central lung squamous cell carcinoma and squamous cell epithelial precancerous lesions can timely and reasonable treatment be carried out. Meanwhile, if the squamous epithelial precancerous lesions can be treated by minimally invasive treatment technique under bronchoscopy before they progress into squamous cell carcinoma [19–20], the prognosis of patients will be further improved [21–22].

CT-occult central lung lesions is reported in the literature as early as 1998, and its early diagnosis is still puzzling clinical workers today [23]. Related study has shown that the occurrence of squamous cell carcinoma is a group of continuous changes from squamous cell epithelial dysplasia, from mild dysplasia to invasive carcinoma takes about 6 years [24], and more than 50% of in-situ cancers can be transformed into invasive carcinoma within 30 months [25]. Such pathological evolution makes early diagnosis of central squamous cell carcinoma possible. Since 2005, China has continuously carried out a number of lung cancer screening programs based on lung CT examination. Most of the patients in this study, especially the high-risk groups with a history of heavy smoking, have received at least one lung CT examination for various causes or lung cancer screening programs in the past 5 years. In this study, it is suggested that most mucosal epithelial lesions in the central airway are underdiagnosed due to CT-occult, and such lesions are not diagnosed for the first time until they results in respiratory tract related symptoms and could be detected by CT examination. This is the main reason for the large diameter and late stage of central lung squamous cell carcinoma at diagnosis. This reflects the insufficiency of CT examination in the early diagnosis of central lung squamous cell carcinoma and squamous epithelial precancerous lesions.

CT is the main means of lung cancer detection, in order to realize the early diagnosis of central airway disease, researchers in various countries have conducted a number of studies on CT. At present, 3D-CT can only achieve the localization and navigation of visible tumors in the lungs, and can not achieve effective diagnosis of early mucosal epithelial lesions in the central airway [26–28]. In recent years, studies based on CT radiomics have been widely carried out. This research team has also used this technology to process CT images to predict the occurrence of CT-occult lesions in central airway, but the effect is still not satisfactory [29]. At present, the early diagnosis of CT-occult lesions can only rely on bronchoscopy, related study has shown that bronchoscopy with autofluorescence and fluoroscopy detects precancerous bronchial lesions located at the level of the bronchial tree [30]. Related research also shows that vascular patterns as visualized by narrow-band imagine also demonstrate acertain predictive role for the histological types of central lung cancer. It also has a high diagnostic value for early in situ squamous cell carcinoma [31].

130 patients with CT-occult lesions and 583 (583/604,96.52%) patients with central lung squamous cell carcinoma undergoing surgical treatment are middle-aged and elderly men with a history of heavy smoking. Meanwhile, such data is in line with the age requirements of many international lung cancer screening programs, and people aged 55–75 are the key population for lung cancer screening [32]. Therefore, for middle-aged and elderly men with heavy smoking, further bronchoscopy is very necessary when no abnormal findings are found in the central airway examined by lung CT.

The study still has some limitations, firstly, the study is a single-center study, and secondly, the patient data is from 2014 to 2018. Despite these limitations in this study, Zhejiang Cancer Hospital, as a regional cancer medical center, has a fixed source of patients, and the lung cancer screening based on lung CT examination has not changed since 2014, meanwhile, Zhejiang Province is a province with good economy, and the coverage of lung cancer screening is wide.so the data selected by this study is of certain research value.

## Conclusions

This study indicates that the current lung CT examination of lung cancer is indeed insufficiency for the early diagnosis of central squamous cell carcinoma and squamous epithelial precancerous lesions. Further bronchoscopy in middle-aged and elderly men with a history of heavy smoking can make up for the lack of routine lung CT examination.

## Data Availability

The datasets used and/or analysed during the current study are available from the corresponding author on reasonable request.
